# 

**Published:** 1897-02

**Authors:** 


### CONTENTS.

ORIGINAL COMMUNICATIONS—

THE RELATION OF RHEUMATIC DIATHESIS TO COMMON THROAT INFLAMMATIONS. By James Edward Newcomb, M.D., New York.......... 793
ACUTE CEREBRAL PALSIES IN CHILDREN. By S. G. Courtney Pinckney, M.D., Atlanta, Ga.......... 799
REMARKS ON PULMONARY TUBERCULOSIS. By W. S. Kendrick, M.D., Atlanta, Ga.... 806
PUERPERAL CONVULSIONS, AND THEIR PREVENTION. By John N. Upshur, M.D., Richmond, Va.......... 810
THE CLINICAL EXAMINATION OF SPUTUM. By James I. Johnston, M.D., Pittsburg, Pa. 815

SOCIETY REPORTS—

SECTION ON OPHTHALMOLOGY, COLLEGE OF PHYSICIANS OF PHILADELPHIA.......... 820

CORRESPONDENCE—

MEDICAL ASSOCIATION OF GEORGIA.......... 826

"GOD AND THE DOCTOR WE ALIKE ADORE".......... 827

EDITORIAL—

MEDICINE IN 1896.......... 828
THE COLONIZATION OF EPILEPTICS.......... 831
THE FAMINE AND PLAGUE IN INDIA.......... 832

SELECTIONS AND ABSTRACTS.......... 835
MEDICAL ITEMS.......... 855
BOOK REVIEWS.......... 861
INDEX TO VOLUME XIII.......... 865
CONTRIBUTORS TO VOLUME XIII.......... 871
MEDICAL ITEMS—CONTINUED.......... x, xii, xiv, xv
ADVERTISERS' INDEX TO ADVERTISEMENTS.......... xviii
CLASSIFIED INDEX TO ADVERTISEMENTS.......... xxxiii

